# F44. AN ADD-ON TRIAL WITH N-ACETYL-CYSTEINE (NAC) IN EARLY PSYCHOSIS PATIENTS: TOWARDS BIOMARKER GUIDED TREATMENT

**DOI:** 10.1093/schbul/sby017.575

**Published:** 2018-04-01

**Authors:** Philippe Conus, Margot Fournier, Lijing Xin, Martine Cleusix, Philipp S Baumann, Carina Ferrari, Ann Cousins, Luis Alameda, Mehdi Gholam-Razaee, Philippe Golay, Raoul Jenni, Tsung-Ung Wilson Woo, Matcheri Keshavan, Chin B Eap, Joanne Wojcik, Michel Cuenod, Thierry Buclin, Rolf Gruetter, Larry Seidman, Kim Do

**Affiliations:** 1 Lausanne University; 2 Lausanne University Hospital; 3 Ecole Polytechnique Fédérale de Lausanne; 4 Centre Hospitalier Universitaire Vaudois, University of Lausanne; 5 Beth Israel Deaconess Medical Center, Harvard Medical School; 6 Harvard Medical School, McLean Hospital; 7 Harvard Medical School; 8 Beth Israel Deaconess Medical Center; 9 Ecole Polytechnique Fédérale de Lausanne, Lausanne University Hospital

## Abstract

**Background:**

Oxidative stress, coupled with dysregulation of inflammation, NMDAR and dopamine, is involved in schizophrenia (SZ) pathophysiology. Earlier add-on clinical trials showed in chronic SZ patients that NAC, a precursor of glutathione (GSH), an important cerebral antioxidant, improved negative symptoms, mismatch negativity and local synchronization. We hypothesized that NAC at an earlier stage of illness would have a greater impact.

**Methods:**

Early psychosis patients (EP, less than 5 years of illness, N=63; NAC=32, placebo=31) were supplemented with NAC (2.7g/day, 6 months) in a double-blind randomized placebo-controlled trial. Outcome measures: PANSS and neurocognition (MATRICS Consensus Cognitive Battery; n=36); quantification of medial prefronfal cortex glutathione (GSHmPFC) by 1H-magnetic-resonance-spectroscopy, of white matter diffusion properties estimated by generalized fractional anisotropy (gFA) computed from diffusion spectrum imaging (DSI), of blood cells GSH (GSHBC) and GSH peroxidase activity (GPxBC) at start and end of trial

**Results:**

While PANSS negative and positive were not affected by NAC, NAC improved Processing Speed (NAC > Placebo; F(1, 30)=5.849, p=.022), favoring 2 of 3 processing speed tasks (Trail Making A, F(1, 30)=4.279, p=.048 & Verbal Fluency, F(1, 30)=5.749, p=.023). GSHmPFC (+23%, p=0.005) and GSHBC (+19%, p=0.05) were increased following NAC treatment. In patients with high-baseline GPxBC (>22.3U/gHb), subgroup explorations revealed an improvement of PANSS positive compared to placebo (p=0.02). The change of PANSS positive correlated negatively with that of GPxBC activity, showing that the improvement paralleled the restoration of redox status. NAC group showed 11% increase in fornix white matter integrity as measured by gFA, correlating with an increase in GSHmPFC over the 6-months period.

**Discussion:**

This is the first clinical trial assessing the impact of NAC treatment in a sample of EP and the potential predictive role of peripheral biomarkers of redox dysregulation. The hypothesis that NAC would be beneficial to negative symptoms in EP was not confirmed in this small sample, most likely in reason of their very low level at baseline. The NAC induced GSHmPFC increase demonstrates its target engagement. NAC improved Processing Speed showing a therapeutic enhancement of cognitive functions. Most importantly, NAC improved fornix integrity, in association with brain GSH elevation, demonstrating for the first time that a redox regulator can enhance structural connectivity. Peripheral redox status allows identifying a subgroup of patients with improved positive symptoms. Future biomarker guided antioxidant interventions in larger EP samples should replicate these findings.

